# GADNN: a revolutionary hybrid deep learning neural network for age and sex determination utilizing cone beam computed tomography images of maxillary and frontal sinuses

**DOI:** 10.1186/s12874-024-02183-9

**Published:** 2024-02-27

**Authors:** Omid Hamidi, Mahlagha Afrasiabi, Marjan Namaki

**Affiliations:** 1 Department of Science, Hamedan University of Technology, Hamedan, Iran; 2 Department of Computer Engineering, Hamedan University of Technology, Hamedan, Iran

**Keywords:** Paranasal sinuses, Dentistry, Artificial intelligence, Machine learning, Genetic algorithm, Forensic dentistry, Forensic science

## Abstract

**Introduction:**

The determination of identity factors such as age and sex has gained significance in both criminal and civil cases. Paranasal sinuses like frontal and maxillary sinuses, are resistant to trauma and can aid profiling. We developed a deep learning (DL) model optimized by an evolutionary algorithm (genetic algorithm/GA) to determine sex and age using paranasal sinus parameters based on cone-beam computed tomography (CBCT).

**Methods:**

Two hundred and forty CBCT images (including 129 females and 111 males, aged 18–52) were included in this study. CBCT images were captured using the Newtom3G device with specific exposure parameters. These images were then analyzed in ITK-SNAP 3.6.0 beta software to extract four paranasal sinus parameters: height, width, length, and volume for both the frontal and maxillary sinuses. A hybrid model, Genetic Algorithm-Deep Neural Network (GADNN), was proposed for feature selection and classification. Traditional statistical methods and machine learning models, including logistic regression (LR), random forest (RF), multilayer perceptron neural network (MLP), and deep learning (DL) were evaluated for their performance. The synthetic minority oversampling technique was used to deal with the unbalanced data.

**Results:**

GADNN showed superior accuracy in both sex determination (accuracy of 86%) and age determination (accuracy of 68%), outperforming other models. Also, DL and RF were the second and third superior methods in sex determination (accuracy of 78% and 71% respectively) and age determination (accuracy of 92% and 57%).

**Conclusions:**

The study introduces a novel approach combining DL and GA to enhance sex determination and age determination accuracy. The potential of DL in forensic dentistry is highlighted, demonstrating its efficiency in improving accuracy for sex determination and age determination. The study contributes to the burgeoning field of DL in dentistry and forensic sciences.

## Introduction

In contemporary contexts, the determination of identity factors such as age and sex has gained considerable significance due to its applications in both criminal and civil cases [1, 2]. These factors play a crucial role in profiling individuals within forensic analyses. For gaining insight into an individual’s profile, forensic medicine employs methods such as general examination, radiography, and supplementary biological tests [3]. In certain accidents, where the use of conventional methods is greatly restricted and establishing a positive identification is not feasible, the examination of dental and pelvic bone structures emerges as a reliable method [2, 4]. However, in specific incidents such as severe burns and trauma, where the possibility of using these methods is also unavailable, there is a need to utilize structures like the paranasal sinuses, including the frontal and maxillary sinuses, known for their resistance to external trauma [2–6].

The anatomical structures of the body, including craniofacial structures, may differ in dimensions between men and women [7, 8]. Hence, this dimensional disparity can also be assessed within the paranasal sinus structures, and if a variance exists, it can be employed for sex determination. In the realm of forensic medicine, age determination at the time of death aids in establishing the profiling of the deceased individual. This age determination is also valuable for individuals migrating to foreign countries or presenting false identities [9, 10]. Although there is no consensus on the relationship between age and the volume of paranasal sinuses in different studies [11–14], some studies have shown that there is a relationship between the volume of sinuses and increasing age [11]. Further studies on this topic can provide valuable information to forensic medicine (for the purpose of more accurate age determination). The maxillary sinus is the first paranasal sinus to form. It is located in the left and right maxillary bones. It is said that this sinus forms in the late second month of pregnancy, and its growth and development are complete between the ages of 18 and 20 years [15]. The frontal sinuses are also air-filled spaces that usually start to grow at the age of 2–3 years, and this process is complete at the age of 20 years. This sinus is also as resistant to environmental damage as the maxillary sinus, so it can be used in profiling individuals [4, 5].

There are many different methods that can be used to determine sex and age using cranio-facial structures, such as measuring dry skulls [16], conventional radiographs [17], and computed tomography [18]. CBCT, or cone-beam computed tomography, is a type of high-resolution imaging of cranio-facial structures [19] that was introduced in the mid-1990s.

The introduction of CBCT dramatically transformed oral and maxillofacial radiology, offering detailed 3D views with significantly less radiation than medical CT [20]. CBCT images are now widely used in diagnostic and three-dimensional reconstruction studies for surgical, orthodontic, and dental implant treatments [2, 3]. CBCT images are now widely used in diagnostic and three-dimensional reconstruction studies for surgical, orthodontic, and dental implant treatment. CBCT archives in healthcare centers can be used to study the dimensions and volume of paranasal sinuses and to assess their relationship with various factors, including age and sex. There have been conducted studies on the relationship between the measurement of frontal or maxillary sinus dimensions and age and sex determination and their findings showed that the dimensions of both frontal or maxillary sinus differs significantly by sex [2, 4, 5, 18]. However, most of these studies applied classical statistical methods and developed models using logistic regression and/or discriminant analysis. The total accuracies reported by these studies vary between 71 and 76%. Therefore, creating innovative methods using state-of-the-art methods is of great importance.

Deep learning (DL), as a subset of artificial intelligence, has received much attention in medical fields, including forensic sciences, in the last few years. DL’s algorithms model high-level concepts by learning mathematical relationships (linear and non-linear) between input and output layers at different levels and layers. The task of the intermediate layers of deep models, which are located between the input and output layers, is to identify data patterns. In order to train DL models, one can use all four machine learning training approaches, i.e., supervised learning, unsupervised learning, semi-supervised learning, and reinforcement learning [21]. The outstanding performance of DL methods has been confirmed by various studies in different fields. DL is a technology that self-learns from data, and it has been shown by studies that DL provides more effective and prominent results compared to other algorithms like decision trees, artificial neural networks, and Naïve Bayes [22]. A DL network is a technique that applies the properties of artificial neural networks where neurons are linked to each other through numerous layers of representation [23]. The representation of data is learned by the DL technique by expanding the level of consideration across levels, which leads to enhanced accuracy.

The significance of DL in dentistry is on the rise as it strives to alleviate the burdens on professionals dealing with ever-expanding datasets. This technology enhances efficiency in processing and reporting data while elevating the accuracy of interpretation. Numerous dental specialties, such as cariology [24], endodontology [25], periodontology [26], and forensic dentistry [27] have displayed promising outcomes through diverse applications of DL techniques. However, to the best of our knowledge, there is no study that has used DL methods for determining sex or age. Therefore, this study aimed to develop a supervised DL model to evaluate the validity of frontal and maxillary sinus dimensions in age and sex determination based on cone beam computed tomography images. Previous studies have shown that the use of evolutionary algorithms in the bodies of other algorithms usually improves their performance [28–31]. We additionally combined the DL approach with a heuristic optimization technique, the genetic algorithm (GA), to introduce an innovative method for feature selection. Therefore, an attempt was made to achieve this goal by using the genetic algorithm.

## Materials and methods

### Dataset and data preprocessing

In this study, a total of 240 CBCT images of 240 Iranian patients (including 129 females and 111 males) were obtained from the CBCT archive of the Department of Oral and Maxillofacial Radiology of the School of Dentistry for various reasons. Their ages ranged from 18 to 52. The following patients were excluded from the study: Congenital craniofacial disorders; orthognathic surgery patients; patients with facial asymmetry; patients with one or more missing posterior teeth, including the first and second molars in the upper jaw (contrary to the first and second molars, the roots of the maxillary first and second premolars have little connection with the maxillary sinus floor; therefore, patients who had lost maxillary premolars were not excluded from the study); patients with frontal sinus aplasia; and CBCT images with evidence of maxillary and frontal sinus pathologies, such as mucosal thickening, sinusitis, and odontogenic cyst or tumor. All CBCT images were obtained using the Newtom3G device (Verona, Italy). The exposure parameters were: kVp 110, mA 3, exposure time 1.8 s, and field of view 15 × 15 cm. Then, this data is imported into the software ITK-SNAP 3.6.0 beta. Using this software, the data, including the length, width, height, and volume of the frontal and maxillary sinuses on the right and left sides, were collected (Table 1).

**Table 1:** Variables used in the model

Variable	Description	abbreviations	Measurement method	Role
Age	Age of participants in year	-	Medical Records	Output
Sex	Patients’ sex status	-	Medical Records	Output
Frontal sinus volume	Lucent space (filled with air) inside the frontal bone	FSV	CBCT Images	Input
Volume of right and left maxillary sinus	Lucent space (filled with air) inside the maxilla bone	RMSV/LMSV	CBCT Images	Input
Length of the frontal sinus	The greatest distance between the most anterior point of the sinus and its most posterior point	FSL	CBCT Images	Input
Frontal sinus width	The greatest distance of the outermost point of the sinus from its innermost point	FSW	CBCT Images	Input
Height of the frontal sinus	The greatest distance of the highest point of the roof of the sinus from the lowest point of its floor	FSH	CBCT Images	Input
Length of right and left maxillary sinus	The greatest distance between the most anterior point of the sinus and its most posterior point x	RMSL/LMSL	CBCT Images	Input
Right and left maxillary sinus width	The greatest distance of the highest point of the roof of the sinus from the lowest point of its floor	RMSW/LMSW	CBCT Images	Input
Height of right and left maxillary sinus	The greatest distance of the highest point of the roof of the sinus from the lowest point of its floor	RMSH/LMSH	CBCT Images	Input

The prediction of the two variables of age and sex based on other characteristics was the goal of this research. An important point to consider in modeling this issue is the difference in nature of the variables that are being investigated. Sex is a binary variable that is easily used to predict different classification models. Age is a continuous variable that is usually considered a regression problem. We considered age as both continuous and categorical outcome and handle it in two scenarios. In the first scenario, people are divided into 4 age groups to determine age. So, our problem became a multi-class classification problem. The minimum age in the data set was 18 years, and the maximum age was 52 years. By trial and error, the best grouping for the data was given in Table 2. In the second scenario, age was considered in its original format (a continuous outcome).

**Table 2:** Age groups and the distribution of the age before and after implementing SMOTE technique

Group Labels	Age groups	Total sample size before SMOTE	Total sample size after SMOTE	Training set sample size	Testing set sample size
A	25 − 18	39	90	77	13
B	33 − 26	70	90	66	24
C	41 − 34	90	90	79	24
D	50 − 42	40	90	66	11

### The proposed method

**Fig. 1:**
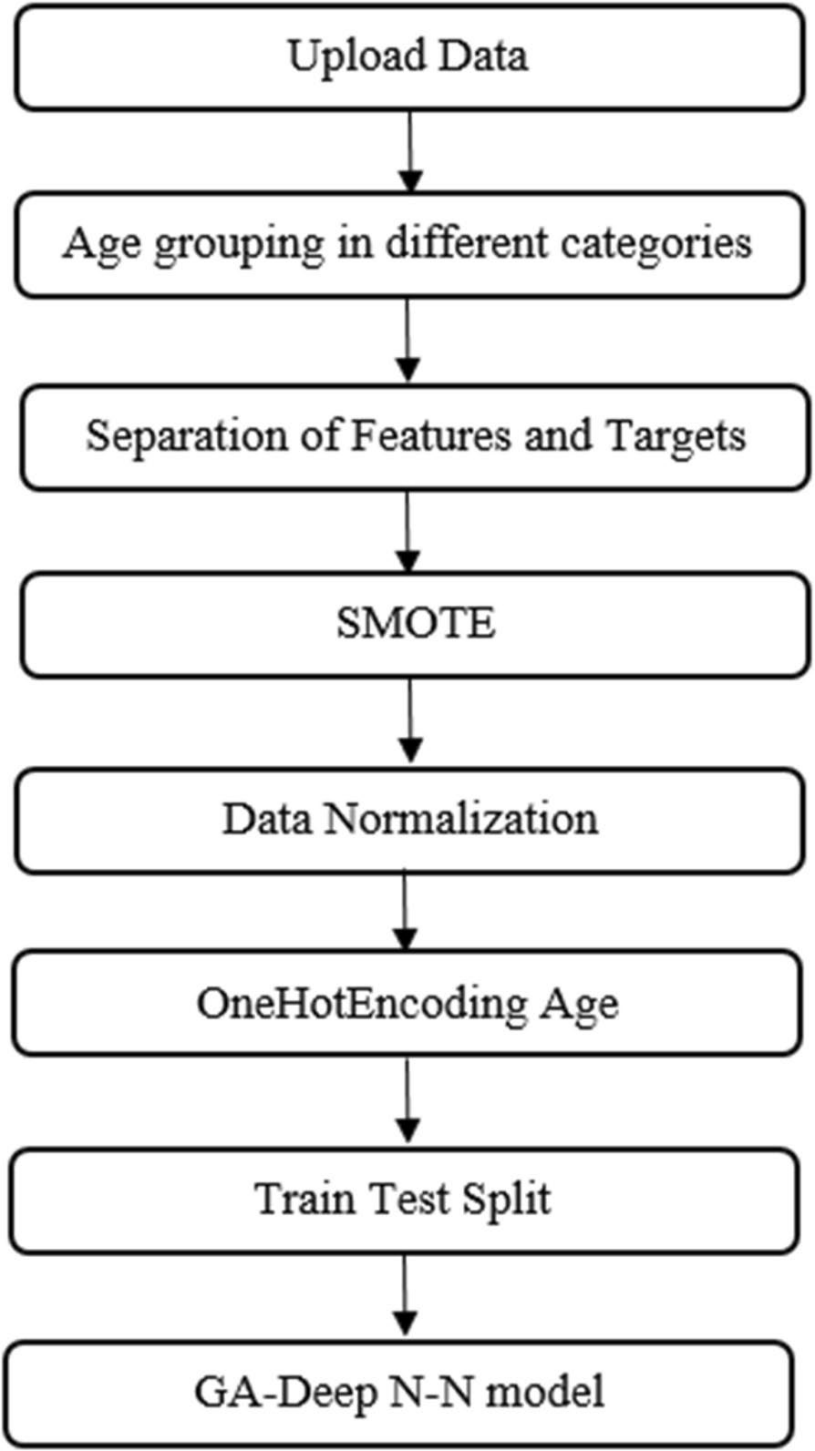
The flowchart of the proposed method for age classification

The proposed method is shown in Fig. 1. In the first step, after uploading the data set, age grouping was done in different age categories. Then the characteristics and target variables (age and sex) have been separated. In order to balance the number of samples across the available age groups, random sampling was performed based on the available data using the synthetic minority oversampling technique (SMOTE) [32]. After normalizing the input data (features), the algorithm was implemented with a DL model. To implement the deep neural network (DNN) model, it was necessary to code the age groups into binary codes. So, for this purpose, the OnHotEncoding technique was used converting categorical variables into binary vectors [33]. And finally, after determining the training data and testing, the learning of the model was done. In the following, the steps of the proposed method are explained:

Since in this study, classes with a 7-year interval were considered to determine the age of people and the number of samples in the classes was unbalanced due to the limited number of samples, in order to balance the data in each class, the synthetic minority oversampling technique (SMOTE) method was used for sampling [32]. Considering the number of examples in the group that has the maximum number of members, the function starts creating examples similar to those in other classes. Considering that 20% of the entire data set was randomly assigned to the test data, in order to avoid an unbalanced distribution of different age classes in the training and testing data, the SMOTE method was used to generate samples similar to the data in different age classes to balance the data. Table 2 also displays the distribution of age for each age class before and after applying SMOTE. The initial dataset contained 239 samples. After applying SMOTE for oversampling, the data was expanded to 380 samples. This resulted in training and testing sets split 80/20. The testing set comprised 13 individuals aged 18–25, 24 aged 26–36, 24 aged 37–41, and 11 aged 42–50. These age groups were chosen to ensure adequate representation in each category. In the next step, the MinMaxScaler method was used to normalize the values of the features. Then, the groups were coded using the OneHotEncoding method to classify the data in perceptron and DNN models.

### Deep learning

DL is a new branch of machine learning algorithms that models high-level concepts by learning mathematical relationships existing between input and output layers, at different levels and layers. The structure of DL models consists of several layers, including the initial and final layers, which are considered the input and output layers of deep models, respectively. These layers are responsible for receiving input data and providing the final output of the model. Moreover, the task of the intermediate layers of deep models, located between the input and output layers, is to identify data patterns. In order to train DL models, one can use all four machine learning training approaches, i.e., supervised learning, unsupervised learning, semi-supervised learning, and reinforcement learning [21].

A DNN is a network that has more than one intermediate layer and it is designed to discover complex patterns in data. DNNs are used for applications such as classification and clustering, and because of their structure, they also include reinforcement learning. In this study, a DNN was used to achieve the desired results [34].

### Genetic algorithm

GA is a family of computational models inspired by the concept of evolution. The basis of GA is the law of natural selection. In such a way that only samples of each generation are able to produce the generation that has the best characteristics, and the others gradually disappear over time. The production of new generations is done by the combination of good chromosomes from each generation. Sometimes mutations also occur on the chromosomes, which, in some cases, lead to better chromosomes in the new generation. GA encodes the candidate solutions to solve the problem in a structure called the chromosome. In the search process to find the optimal solution, first a set or a population of initial solutions is generated. Then, in successive generations, a set of modified solutions is produced by the reproductive operators. The initial solutions usually change in such a way that in each generation, the population of solutions converges towards the optimal solution. Then, it is evaluated by the fitness function, and if the termination conditions of the algorithm are met, the algorithm is terminated. The main members of GA are the fitness function, selection operators, and reproduction operators. The representation structure of chromosomes is defined as binary, true, correct, etc., depending on the type of problem [35].

In the present study, to achieve higher accuracy in predictions, GA was used to select the best features from the dataset. Therefore, the type of representation of chromosomes was considered binary. The fitness function was the accuracy of the DNN. In the following section, the DNN architecture is explained.

### GADNN model and the architecture of the network

**Fig. 2:**
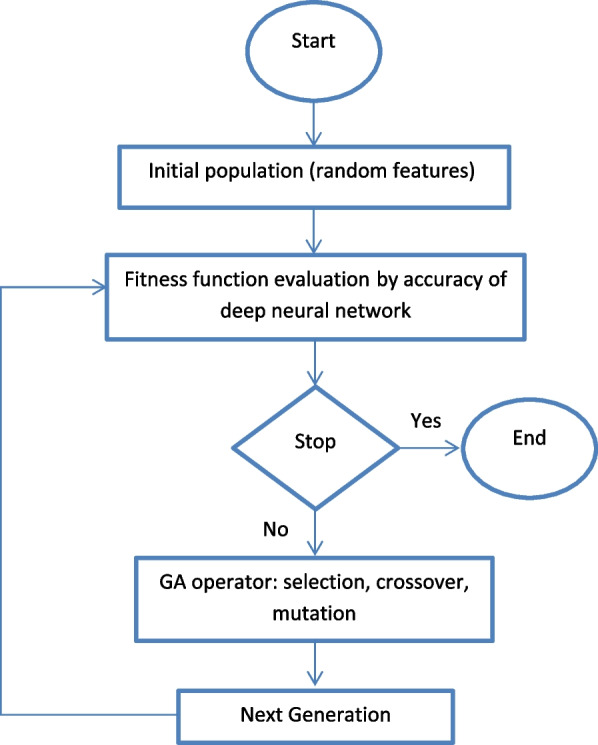
Overview of the GADNN model

The model used in this research is called GADNN (genetic algorithm-based Deep Neural Network). The main problem solving model in this research was the supervised DNN, which selected the appropriate features. Also, a combination of GA and DNNs was used. Figure 2 shows the flowchart of the GADNN model. In this way, the fitness function of the GA was the DNN designed for the at-hand problem.

**Fig. 3:**
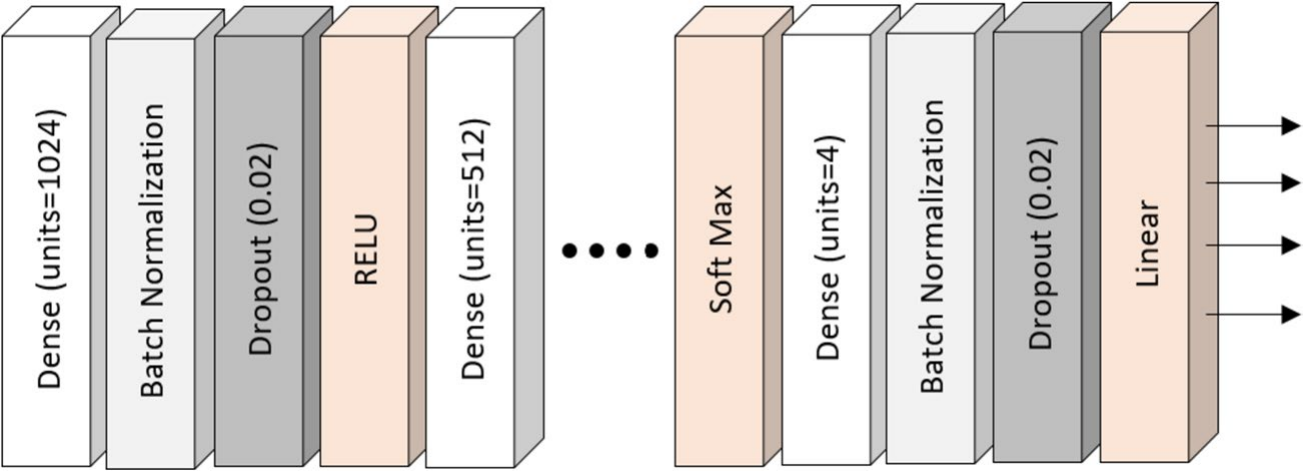
*N*etwork architecture

Figure 3 shows the architecture of the network. This network consisted of 9 main layers. The first layer had 1024 neurons and 12 inputs, equal to the number of features. The second layer had 512 neurons, the third layer had 256 neurons, and the fourth, fifth, sixth, seventh, eighth, and ninth layers had 128, 64, 32, 16, 8, and 4 neurons, respectively. Since the last layer shows the final output of the network, a separate branch was considered for each age group. After each main layer, a batch normalization layer was placed in order to accelerate and stabilize the DNN. This layer performs standardization and normalization of the input of each layer. Dropout layers have also been used to avoid the problem of overfitness after each layer. The activation function of the first to seventh layers was ReLU (rectified linear unit), and the activation function of the eighth layer was Softmax. Also, for the output layer, a linear activation function was considered.

$$Relu: f\left(x\right)=\text{m}\text{a}\text{x}(x,0)$$

$$softmax:\text{f}\left({x}_{i}\right)= \frac{{e}^{{x}_{i}}}{\sum _{j}{e}^{{x}_{j}}}$$

$$Linear: f\left(x\right)=x$$

The mean square error (MSE) was considered the network’s loss function, and stochastic gradient descent was considered the model optimizer. Training and testing data were set at 100 epochs. Since the age values were coded into binary values before running the model, but the output of the neural network was based on age groups, to calculate the model evaluation criteria, the predicted values were coded into binary values. In all models, 80% of the data were assigned to the training data and 20% to the testing data.

### Evaluation of models

In order to evaluate the models, F1 score, precision, and recall criteria have been used. Below is how to calculate these criteria [36]. The first and most important criterion is the accuracy or correctness of the model, which is equal to the number of correctly predicted cases over the total number of predictions. If the prediction classes are divided into positive and negative classes, TP represents the number of true positive predictions, TN represents the number of true negative predictions, FN represents the number of false negative predictions, and FP represents the number of false positive predictions. The formulas are as follows:

$$Accuracy= \frac{TP+TN}{TP+TN+FP+FN}$$

$$Recall= \frac{TP}{TP+FN}$$

$$Precision= \frac{TP}{TP+FP}$$

$$F1=2* \frac{Precision*Recall}{Precision+Recall}$$

We also used root mean square error and R-squared criteria for evaluating the methods in predicting age as a continuous outcome. RMSE (Root Mean Square Error) is a common metric used to measure the accuracy of regression models. A lower RMSE indicates a more accurate model. R-squared (R^2^) is a measure of how well the model fits the data. A higher R-squared indicates a better fit with a maximum of 1.

### Implementation of the models

In this study, four machine learning models, including logistic regression (LR), random forest (RF), multi-layer perceptron (MLP) and DL were considered. These models were trained to predict age and sex. For machine learning models, some operations, such as coding and feature selection, have not been done. First, it was tried to solve the problem with regression, so the logistic regression model was used to predict age. The results indicated that this model was not a suitable solution for the problem. Therefore, the MLP model was used. MLP consisted of three layers. The input layer provided the input data, the hidden layer computed the complex connections between the network, and the output layer obtained the results. To run the model, the ReLU activation function was used, and the number of layers was set to 150, 100, and 50. The results were better than the previous model but not convincing. Therefore, the third model, RF, was used to predict age. In this model, using the k-fold method, data and features were selected greedily and randomly. The performance of this model was much better than the previous two models. In order to check and achieve higher accuracy, a DNN model was used.

## Results

The results of the model implementation showed that DNNs had higher accuracy than other models. The output of the GADNN model led to the elimination of three features: FSW, FSV, and LMSW. Next, DNNs and GA were examined. Also, better results were obtained by using the GADNN model and removing the three features of FSW, FSV, and LMSW (see Table 1 for the complete name).

Table 3 presents the evaluation metrics of various models used for age determination. The results reveal a varying range of precision scores for different age categories: 0.28 to 0.50 for LR, 0.27 to 0.48 for MLP, 0.38 to 0.65 for RF, 0.14 to 0.57 for DL without SMOTE augmentation, 0.46 to 0.84 for DL with SMOTE augmentation, and 0.44 to 0.83 for GADNN. GADNN consistently outperforms the other methods in terms of precision. The recall values for different age categories also exhibit a range: 0.10 to 0.56 for LR, 0.10 to 0.61 for MLP, 0.25 to 0.83 for RF, 0.12 to 1.00 for DL without SMOTE, 0.38 to 0.87 for DL with SMOTE, and 0.50 to 0.89 for GADNN. Notably, GADNN and DL with SMOTE achieve consistently high recall scores. Similarly, the F1-Score metrics vary across the different models: 0.15 to 0.53 for LR, 0.15 to 0.54 for MLP, 0.30 to 0.73 for RF, 0.13 to 0.57 for DL without SMOTE, 0.41 to 0.80 for DL with SMOTE, and 0.47 to 0.82 for GADNN. GADNN and DL with SMOTE consistently demonstrate superior F1-Scores. In summary, the performance of different models on age determination tasks varies significantly. GADNN and DL with SMOTE consistently outperform the other models, showcasing superior precision, recall, and F1-Score metrics. The RF model ranks second in overall performance.

**Table 3:** The results of evaluation of machine learning and deep neural network models for determining age group

Method	Age Groups	Precision	Recall	F1-Score	Accuracy
Logistic Regression with SMOTE^a^	a	0.50	0.56	0.53	0.36
	b	0.28	0.31	0.29	
	c	0.29	0.10	0.15	
	d	0.33	0.50	0.40	
Multi-layer Perceptron with SMOTE	a	0.48	0.61	0.54	0.38
	b	0.27	0.25	0.26	
	c	0.29	0.10	0.15	
	d	0.37	0.56	0.44	
Random Forest with SMOTE	a	0.65	0.83	0.73	0.57
	b	0.60	0.56	0.58	
	c	0.38	0.25	0.30	
	d	0.57	0.67	0.62	
Deep Learning without SMOTE	a	0.40	1.00	0.57	0.37
	b	0.25	0.33	0.29	
	c	0.57	0.32	0.41	
	d	0.14	0.12	0.13	
Deep Learning with SMOTE	a	0.59	0.87	0.70	0.62
	b	0.50	0.47	0.48	
	c	0.46	0.38	0.41	
	d	0.84	0.75	0.80	
GADNN^b^ with SMOTE	a	0.76	0.89	0.82	0.68
	b	0.67	0.67	0.67	
	c	0.44	0.50	0.47	
	d	0.83	0.65	0.73	

^a^the synthetic minority oversampling technique; ^b^Genetic algorithm based deep neural network model

**Fig. 4:**
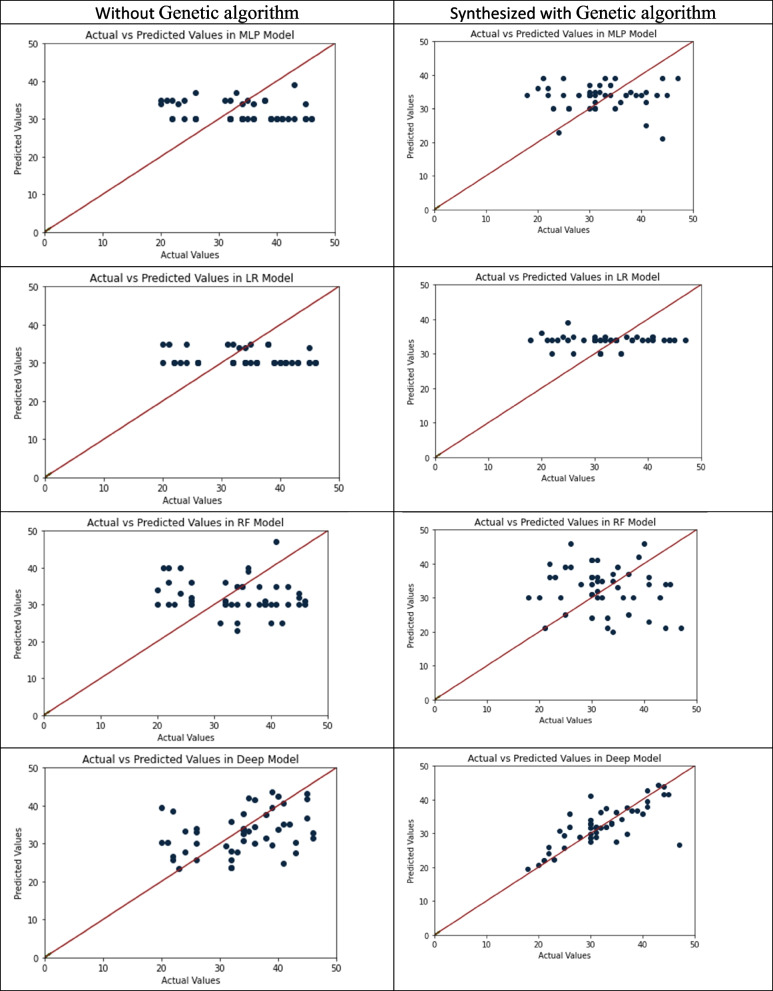
The scatter plots of the observed versus predicted values of age variable by various machine learning and deep learning methods with and without genetic algorithm

In addition to multi-class problem of age prediction, we examined the continuous age outcome by treating age as its original scale and running regression versions of machine learning and deep neural network models. The results were presented in Table 4; Fig. 4, which both showed the performance of the models with and without GA augmentation. According to Table 4, the GADNN model with GA augmentation consistently outperformed the other models, achieving the lowest RMSE value (4.62) and the highest R^2^ value (0.92). Furthermore, Fig. 4 graphically depicts the observed and predicted age values for various methods. The GADNN models exhibited the most consistent agreement between the observed and predicted values and as can be seen there is a strong correlation between them.

**Table 4:** Comparative evaluation of machine learning and deep neural network models for determining age (continuous outcome)

Synthesized	Model	RMSE	R^2^
Without Genetic algorithm	Multi-layer Perceptron	8.919641	0.03
	Logistic Regression	8.672946	0.01
	Random Forest	9.697422	0.09
	Deep Learning	7.776246	0.669
With Genetic algorithm	Multi-layer Perceptron	8.13941	0.06
	Logistic Regression	7.28011	0.03
	Random Forest	10.23328	0.11
	GADNN*	4.623851	0.92

^*^Genetic algorithm based deep neural network model

Table 5 presents a comparative analysis of the performance of different models for sex determination. The results revealed a distinct range of various criteria across sex categories. Notably, the GADNN model with SMOTE augmentation consistently outperformed the other models, achieving superior precision scores of 0.83 and 0.89 for male and female, respectively. Moreover, the GADNN model with SMOTE also demonstrated the highest recall values of 0.87 and 0.86 for male and female, respectively. This remarkable performance is further corroborated by the F1-Score metric, where the GADNN model with SMOTE attains the highest values of 0.85 and 0.88 for male and female, respectively. Additionally, the GADNN model with SMOTE achieves the highest accuracy of 0.86, surpassing the performance of other models such as deep learning (accuracy = 0.78) and random forest (accuracy = 0.71).

**Table 5:** The results of evaluation of machine learning and deep neural network models in determining sex

Method	Sex Groups	Precision	Recall	F1-Score	Accuracy
Logistic Regression with SMOTE^a^	Female	0.70	0.55	0.62	0.62
	Male	0.55	0.70	0.62	
Multi-layer Perceptron with SMOTE	Female	0.75	0.62	0.68	0.67
	Male	061	0.74	0.67	
Random Forest with SMOTE	Female	0.75	0.72	0.74	0.71
	Male	0.67	0.70	0.68	
Deep Learning without SMOTE	Female	0.62	0.71	0.67	0.68
	Male	0.75	0.67	0.71	
Deep Learning with SMOTE	Female	0.79	0.81	0.80	0.78
	Male	0.79	0.76	0.78	
GADNN^b^ with SMOTE	Female	0.89	0.86	0.88	0.86
	Male	0.83	0.87	0.85	

^a^the synthetic minority oversampling technique; ^b^Genetic algorithm based deep neural network model

## Discussions

In forensic medicine, age determination at the time of death helps determine the identity of a dead person. Age determination is also helpful for people who immigrate to other countries or have fake identities [1, 2]. Determining sex can also provide useful information for forensic medicine in order to identify people [3]. The paranasal sinuses, like the maxillary and frontal sinuses, are among the resistant structures that can be used to determine the identity of people by measuring their volume and dimensions [4, 5, 15]. CBCT is a type of imaging of craniofacial structures with high accuracy [4, 14] and has good advantages such as reducing the time and amount of radiation, easy application, and high diagnostic speed [5].

In the present study, CBCT samples of people, including 129 women and 111 men with an age range of 18–51 years, were examined. The dimensions and volume of the right and left maxillary sinuses and the frontal sinus were measured and considered inputs of a new approach for age and sex determination. So far, few attempts have been made for sex determination and age determination based on the maxillary and frontal sinuses obtained from CBCT. Most previous studies have used classical statistical models like linear discriminant analysis. Our study is unique as it adapts a state-of-the-art technology called DL. This method has not been much applied as a prediction tool in age and sex determination for the maxillary and frontal sinus parameters. Two other aspects of the novelty of the present study were that we developed a hybrid model based on synthesizing a heuristic technique and DL and used the SMOTE method to deal with the unbalanced data. The new approach was named GADNN. In the present study, some other widely used machine learning methods, including LR, RF, MLP, DL with or without SMOTE, and with or without GA were also employed and compared to determine sex and age using the maxillary and frontal sinus parameters.

Based on the obtained accuracy measures, it was shown that the proposed methods (GADNN) outperformed other methods in age and sex determination with accuracy of 68% and 0.86, respectively, where other approaches achieved an accuracy of, e.g., 36% and 62%. So, the classical logistic regression did not provide poor performance in age and sex determination for our data. DNNs are considered an upcoming technology in many medical fields and industries, including forensic dentistry. DL has several advantages, such as the fact it learns unknown patterns in the data automatically by minimizing errors and can handle complex data. DL also handles the non-linear and complex relationships between inputs and outputs and provides predictive models. Corbella et al. conducted a systematic review study to explore applications of DL in dentistry. They confirmed the outperformance of DLs compared to other machine learning methods.

Several previous studies have confirmed that the application of artificial intelligence (AI) methods in a variety of dentistry situations showed promising performance in the dental caries detection [37], root fractures [38], root morphologies [39], etc. Nevertheless, it is seen that the number of studies for age and sex determination based on cone beam computed tomography images of maxillary and frontal sinuses using AI is limited.

Paknahad et al. conducted a study with the aim of investigating the relationship between the dimensions of the maxillary sinuses obtained from CBCT images and sex determination. The width, length, and height of the maxillary sinuses were measured in the CBCT images of 100 patients (50 men and 50 women). Independent samples t-test and diagnostic tests were used to analyze the measured parameters. The accuracy of the prediction of sex determination was 78% in women and 74% in men, with an average of 76%. However, they have not used a training and testing approach [4]. Choi et al. conducted a study to evaluate the reliability of frontal sinus CBCT images for sex determination. A total of 130 scans (65 men and 65 women) were reconstructed three-dimensionally. Based on a logistic regression analysis, they have shown an accuracy of 80% for sex determination (again without cross-validation) [2]. Urooge et al. conducted a study with the aim of investigating the relationship between the size and volume of the maxillary sinus (MS) and sex determination by CBCT. Bilateral maxillary sinus images (left and right) were obtained for 100 patients (50 women and 50 men), and various parameters (width, length, height, area, perimeter and volume) were measured and evaluated. The comparison between male and female groups did not show any significant difference in the right and left sides regarding the length, height, area, volume, and range of the maxillary sinus. However, the maxillary sinus width had a statistically significant difference. The final result of the analysis shows that the ability of the maxillary sinus to identify sex is 68% in males and 74% in females, with an overall accuracy of 71%. All these studies were based on classical statistical models, and none have used a cross-validation approach to test the performance of the methods for the new data sets. Cross-validation is a fundamental and crucial step of building and evaluating reliable machine learning models. Cross-validation minimizes the chance of overfitting problem, where the model memorizes the training data (inflated performance metrics over training set) but fails to generalize well to unseen data (poor performance). Cross-validation provides more reliable performance estimates by averaging performance across multiple folds, offering a robust estimate of how a model will perform on new data. Moreover, cross-validation can help detect biases within data. Splitting the data into multiple folds can reveal if a model’s performance is heavily influenced by specific subsets of the data, indicating potential biases that can be addressed through data cleaning or augmentation techniques. Therefore, cross-validation is a fundamental principle for building reliable and generalizable machine learning models. Therefore, the findings of the present study can serve as a starting point for developing DL models to automatically and reliably perform age and sex determination in forensic sciences.

There were some limitations in the present study. Firstly, the data we used in this study did not include original CBCT images, and we only had access to the twelve features extracted from the images. Therefore, it is suggested to utilize CBCT images directly to create automatic age and sex detectors based on convolutional neural networks optimized by evolutionary algorithms. Second, the sample size used here was small, and it is suggested to use larger data sets to achieve higher accuracies. However, the strength of this study was to develop a state-of-the-art model based on DL to determine sex and age. This field is an open research area. It is suggested to use transfer learning to solve the problem and analyze the results.

## Conclusions

This study proposed a hybrid algorithm based on the DL and GA approaches for age and sex determination that handled unbalanced groups using the SMOTE technique. Our findings showed that using the SMOTE technique to solve problems with little data can be effective and improve prediction performance. Also, using evolutionary algorithms to achieve higher accuracy is a solution that can help in this matter. The performance of deep networks is also remarkable due to the discovery of complex feature relationships and better learning than other models. Therefore, the proposed method can be useful for detecting sex and age.

### Software

Codes were written in Python and were provided in https://github.com/mariiijan/GA-deep-net-model/tree/main.

## Data Availability

The datasets generated during and analyzed during the current study are not publicly available due to the Hamedan University of Technology restrictions on public sharing data, but are available from the corresponding author upon reasonable request.

## Abbreviations

GADNN: Genetic Algorithm-Deep neural Network
CBCT: Cone-Beam Computed Tomography
LR: Logistic Regression
RF: Random Forest
MLP: Multilayer Perceptron neural network
DL: Deep Learning
SMOTE: The synthetic Minority Oversampling Technique
GA: Genetic Algorithm
ReLU: Rectified Linear Unit
MSE: Mean Square Error
TP: True Positive
TN: True Negative
FP: False Positive
FN: False Negative
